# Variation rs2235503 C > A Within the Promoter of *MSLN* Affects Transcriptional Rate of Mesothelin and Plasmatic Levels of the Soluble Mesothelin-Related Peptide

**DOI:** 10.3389/fgene.2020.00975

**Published:** 2020-08-18

**Authors:** Roberto Silvestri, Perla Pucci, Chiara De Santi, Irene Dell’Anno, Simona Miglietta, Alda Corrado, Vanessa Nicolí, Daniela Marolda, Monica Cipollini, Enrica Pellegrino, Monica Evangelista, Alessandra Bonotti, Rudy Foddis, Alfonso Cristaudo, Stefano Landi, Federica Gemignani

**Affiliations:** ^1^Department of Biology, University of Pisa, Pisa, Italy; ^2^Division of Cellular and Molecular Pathology, Department of Pathology, University of Cambridge, Cambridge, United Kingdom; ^3^Department of Molecular and Cellular Therapeutics, Royal College of Surgeons in Ireland, Dublin, Ireland; ^4^San Raffaele Telethon Institute for Gene Therapy (SR-Tiget), San Raffaele Scientific Institute (IRCCS), Milan, Italy; ^5^Department of Bioscience, University of Milan, Milan, Italy; ^6^Department of Translational Research and of New Surgical and Medical Technologies, University of Pisa, Pisa, Italy; ^7^Institute of Clinical Physiology (IFC), CNR, Pisa, Italy; ^8^Preventive and Occupational Medicine, University Hospital of Pisa, Pisa, Italy

**Keywords:** soluble mesothelin-related peptide, malignant pleural mesothelioma, diagnostic biomarkers, single nucleotide polymorphisms, fluorescent reporter assay, eQTL

## Abstract

Soluble mesothelin-related peptide (SMRP) is a promising biomarker for malignant pleural mesothelioma (MPM), but several confounding factors can reduce SMRP-based test’s accuracy. The identification of these confounders could improve the diagnostic performance of SMRP. In this study, we evaluated the sequence of 1,000 base pairs encompassing the minimal promoter region of the *MSLN* gene to identify expression quantitative trait loci (eQTL) that can affect SMRP. We assessed the association between four *MSLN* promoter variants and SMRP levels in a cohort of 72 MPM and 677 non-MPM subjects, and we carried out *in vitro* assays to investigate their functional role. Our results show that rs2235503 is an eQTL for *MSLN* associated with increased levels of SMRP in non-MPM subjects. Furthermore, we show that this polymorphic site affects the accuracy of SMRP, highlighting the importance of evaluating the individual’s genetic background and giving novel insights to refine SMRP specificity as a diagnostic biomarker.

## Introduction

Malignant pleural mesothelioma (MPM) is a highly aggressive and rare cancer of the pleura triggered by exposure to asbestos and associated with a poor prognosis (Bianchi et al., 2017). The long latency between the exposure to asbestos and the onset of the disease, the unspecific symptoms at the presentation, and the lack of accurate and non-invasive diagnostic tools make the diagnosis quite challenging (Rusch et al., 2012; Bianco et al., 2018). Thus, MPM is often diagnosed at advanced stages, thereby further reducing the limited therapeutic options available and resulting in poor prognostic outcomes. The identification of accurate diagnostic biomarkers, or the optimization of those identified so far, could help to overcome this problem (Sun et al., 2017). To date, one of the most promising biomarkers for the diagnosis of MPM is “soluble mesothelin-related peptide” (SMRP), which is released in the extracellular environment following the proteolytic cleavage of mesothelin (a membrane protein encoded by the *MSLN* gene) (Sapede et al., 2008). The plasmatic concentration of SMRP is easily measurable from blood samples, and it is usually higher in MPM than in non-MPM subjects (either healthy or affected by other respiratory conditions), allowing fair discrimination between these two groups (Gao et al., 2019; Gillezeau et al., 2019). Despite these promising features, several confounding factors can reduce the accuracy of SMRP as a diagnostic biomarker, thereby preventing its employment in the clinical practice. While many studies extensively documented the impact of age, body mass index, glomerular filtration rate (Casjens et al., 2017), and tumor histology on SMRP (Scherpereel et al., 2006), none of them explored the role of the genetic background. However, we believe that the genetic background could play a prominent role in this context. An increasing number of studies, by showing the importance of genetic variations in altering the concentration and the accuracy of several biomarkers, strongly support this idea (Cramer et al., 2003; Gudmundsson et al., 2010; Enroth et al., 2014; Ruggiero et al., 2015; Wang et al., 2018). Furthermore, we showed for the first time that single nucleotide polymorphisms (SNPs) located within the 3′-UTR of the *MSLN* gene could affect the SMRP levels through a miRNA-mediated mechanism (Garritano et al., 2014). Recently, we reported similar results for other variants lying within the *MSLN* promoter (De Santi et al., 2017). Based on these observations, we hypothesized that expression quantitative trait loci (eQTL) for *MSLN* could affect the plasmatic concentration and the accuracy of SMRP. Thus, we assessed the association between SNPs located within the promoter region of *MSLN* and the plasmatic levels of SMRP in a cohort of 72 MPM and 677 non-MPM subjects. We carried out *in vitro* assays to investigate the functional role of these SNPs, and highlighted the relationship between the transcriptional rate of *MSLN* gene and serum concentrations of SMRP. Our results show that evaluating the individual’s genotype to establish personalized cutoff values can increase the diagnostic accuracy of SMRP, thereby helping to promote its translation into clinical practice.

## Materials and Methods

### Selection of the SNPs

Following the hypothesis that the transcriptional rate of the *MSLN* gene could affect the plasmatic concentration of SMRP, we extracted a window of 1,000 base pairs upstream of the transcription starting site (TSS), encompassing the minimal promoter region, using the UCSC genome browser^1^. Within this sequence, we selected those SNPs showing a minor allele frequency (MAF) >0.05 and a significant association with *MSLN* mRNA levels in lung tissues (according to the GTEx Portal V6p^2^). This selection led to the identification of four SNPs worth of further investigations: rs3764247 A > C, rs3764246 A > G, rs2235503 C > A and rs2235504 A > G (Supplementary Table 1). According to 1,000 Genomes^3^, their combination elicits four haplotypes (Table 1), accounting for almost the totality (98.5%) of the genetic variability of the promoter region within Caucasians, and Tuscans.

**TABLE 1:** Number of individuals stratified according to their health status and genotype at single allele/diplotype level. “MPM” represents patients affected by malignant pleural mesothelioma, while “non-MPM” identifies subjects exposed to asbestos (healthy or affected by benign respiratory diseases). Table also shows the variants (rs) that characterize each haplotype.

Haplotypes distribution and sequences						

Distribution among studied population						

Single Alleles	Non-MPM	MPM	rs3764247	rs3764246	rs2235503	rs2235504
Haplotype 1 (H1)	1047	105	A	A	C	A
Haplotype 2 (H2)	209	24	C	G	A	G
Haplotype 3 (H3)	109	13	A	G	C	G
Haplotype 4 (H4)	47	4	C	A	C	A

**Diplotypes**	**Non-MPM**	**MPM**				

H1H1	389	39				
H1H2	152	14				
H1H3	78	11				
H1H4	39	2				

### Genotyping, Haplotype Reconstruction, SMRP Measurements and Association Study

Detailed information about the studied population, DNA extraction, genotyping, and measurement of SMRP levels can be found elsewhere (De Santi et al., 2017). Briefly, we analyzed a total of 677 non-MPM and 72 MPM volunteers. Among the 677 non-MPM, 371 were healthy people, while 306 were patients affected by benign respiratory diseases (BRD). All the subjects were recruited at the University Hospital of Pisa as part of an occupational surveillance program on workers previously exposed to asbestos, as described by Garritano et al. (2014). Blood and serum samples were obtained by venipuncture and store at −80°C. To measure the plasmatic concentration of the SMRP, we employed the Mesomark enzyme-linked immunosorbent assay (Fujirebio Diagnostics, Japan) following the manufacturer’s instructions. We carried out DNA extraction and genotyping using the EuroGOLD Blood DNA Mini Kit (EuroClone, Pero, Italy) and the KASPar PCR SNP genotyping system (LGC Genomics Ltd., Teìddington, Middlesex, United Kingdom), respectively. For this study, we reconstructed the individual haplotypes and diplotypes using PHASE 2.1.1 (Stephens et al., 2001; Stephens and Scheet, 2005) and stratified our cohort according to health status and diplotypes. To precisely assess the effect of each haplotype, we restricted the association study to carriers of the haplotype #1 (e.g., H1H2, H1H3, and H1H4), using the H1H1 homozygotes as reference. The number of subjects excluded from the analysis was minimal (19 non-MPM and 6 MPM) and did not hamper the statistical power of the study.

### Cell Lines

For the functional study, we employed a non-malignant SV40-immortalized epithelial cell line (MeT-5A) and an epithelioid malignant mesothelioma cell line (Mero-14). MeT-5A were purchased from ATCC (American Type Culture Collection, CRL-9444) and cultured in Medium-199 (Gibco, Life Technologies, Monza, Italy) supplemented with 10% fetal bovine serum (FBS), 1% penicillin/streptomycin, 3 nM epidermal growth factor (EGF), 400 nM hydrocortisone and 870 nM insulin. Mero-14 were kindly donated by Istituto tumori of Genova (National Research Council, Genova, Italy) and cultured in Dulbecco’s modified Eagle’s medium (DMEM; EuroClone, Pero, Italy) supplemented with 10% FBS and 1% penicillin/streptomycin. Both cell lines were maintained at 37°C and 5% CO_2_.

### Construction of Plasmids

**FIGURE 1:**
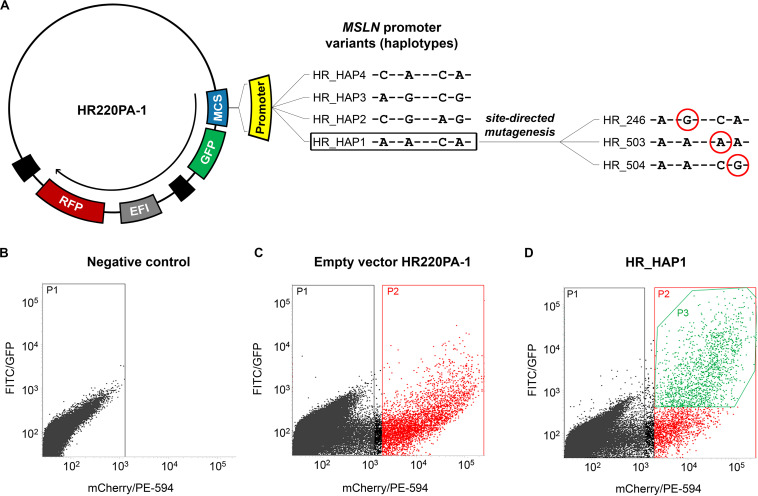
Construction of the plasmids and cells selection. **(A)** scheme of the vectors harboring the four haplotypic variants of *MSLN* promoter (HR_HAP1 to HR_HAP4) upstream of a GFP reporter gene employed in the functional study. The same vectors also harbor an internal control represented by an RFP reporter gene under the control of the elongation factor 1 (EF1) constitutive promoter. HR_HAP1 has been used as a template for a site-directed mutagenesis procedure, to obtain the vectors HR_246/503/504 that have been employed to assess the effect of the single SNPs in altering the GFP expression. The SNPs variants are circled in red. **(B–D)** Graphs showing the fluorescence intensity, at single cell level, measured using FITC/GFP (GFP fluorescence) and mCherry/PE-594 (RFP fluorescence) filters with BD FACSJazz System. **(B)** Untransfected cells (P1) have been used as a control to establish the RFP intensity threshold for the successfully transfected cells. **(C)** Cells successfully transfected (P2) with the empty vector HR220PA-1, expressing only the RFP reporter gene, have been used to determine the average GFP background level. **(D)** The statistical analysis has been restricted to successfully transfected cells that showed a GFP signal above the average GFP background (P3) (HR_HAP1 is reported as an example).

In the first part of this work, we aimed to evaluate the role of the four haplotypes herein identified in affecting the activity of the *MSLN* promoter. Thus, we constructed four vectors to employ in the functional study. Each of these vectors harbored one of the haplotypic variants immediately upstream of a green fluorescent protein (GFP) coding sequence. The same vectors also harbored a red fluorescent protein (RFP) coding sequence controlled by the constitutive eukaryotic promoter EF1 (Figure 1A). For the cloning procedure, we employed the CloneEZ PCR cloning kit (GenScript, Piscataway, United States). We used the HR220PA-1 vector (System Bioscience, Palo Alto, United States) linearized with *Bst*BI (New England BioLabs, Ipswich, United States) and DNA fragments representing the four haplotypic variants of the *MSLN* promoter. Each of these fragments was obtained by PCR using as a template the genomic DNA of four subjects homozygote for one of the four selected haplotypes (now on abbreviated as H1–H4 when referring to the human genomic DNA, or HAP1–HAP4 when referring to the cloned fragment). The resulting vectors were named HR_HAP1 to HR_HAP4. In the second part of this work, we aimed to ascertain the individual role of each of the selected SNPs. To this end, we created three additional vectors, each harboring the uncommon variant of only one of the four SNPs. These vectors were named HR_246, HR_503, and HR_504 and were obtained from HR_HAP1 by site-directed mutagenesis using the Quick Change Lightning Site-Directed Mutagenesis Kit (Agilent). Consistently with the terminology employed in this second phase of the study, since the HR_HAP4 differs from the HR_HAP1 only for the rs3764247, bearing the C-allele instead of the A-allele, we renamed it “HR_247.” Figure 1A shows the details of the changes introduced by the site-directed mutagenesis to generate these additional vectors.

### Fluorescent Reporter Assay

To evaluate the effect of the selected haplotypes and SNPs on the transcriptional activity of the *MSLN* promoter, we employed a fluorescent reporter assay. To this end, 2 × 10^5^ cells/ml were electroporated with 10 μg of plasmid DNA, seeded into a six-well plate, and incubated at 37°C and 5% CO_2_ for 72 h. After the incubation, we harvested the cells by trypsinization and measured the GFP/RFP fluorescence intensity using the BD FACSJazz System (BD Biosciences, Franklin Lakes, United States). We used the untreated sample to set the RFP intensity threshold for the selection of efficiently transfected cells (Figure 1B). Similarly, we used cells transfected with the “empty” HR220PA-1 vector (expressing the RFP but not the GFP reporter gene) to establish the average background signal detected by the FITC/GFP channel (Figure 1C). These two steps allowed us to restrict the statistical analysis only to the cells that were efficiently transfected, and that showed a GFP signal above the average background (Figure 1D; P3). All the electroporation steps were carried out using the Neon Transfection System (Thermo Fisher Scientific, Monza, Italy), and the following parameters: 1,230 V, 30 ms, 2 pulses for MeT-5A and 1,130 V, 30 ms, 2 pulses for Mero-14.

### Statistical Analyses

For the statistical analyses, we employed GraphPad PRISM 7.0 software. We carried out the analysis of variance (ANOVA) for the association study and the multifactor ANOVA (mANOVA) for the functional studies, both followed by Dunnett’s multiple comparison test. To evaluate the diagnostic performance of SMRP along with the optimal cutoff values, we calculated the receiver operating characteristic (ROC) curves employing the same software.

## Results

### Haplotype #2 and Haplotype #3 Are Associated With SMRP Levels *in vivo*

**FIGURE 2:**
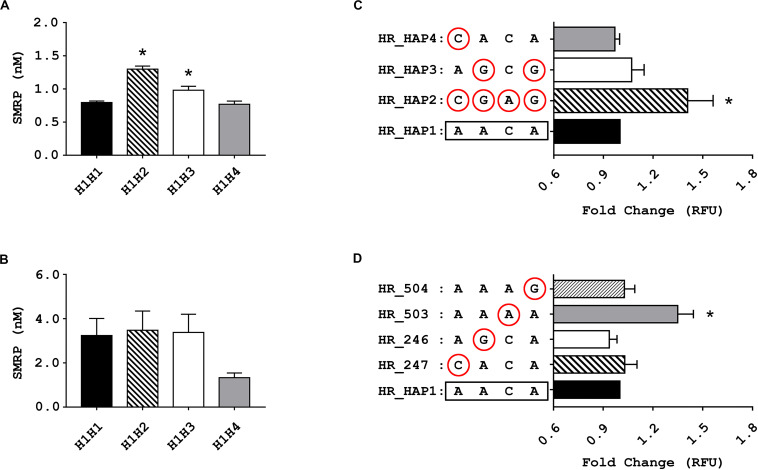
Results of the association and functional studies. **(A,B)** Bar charts showing the association between the genetic variants of *MSLN* promoter and the levels of soluble mesothelin-related peptide (SMRP) measured in blood samples from non-MPM subjects **(A)** and from patients affected by pleural mesothelioma (MPM) **(B)** stratified according to their individual diplotypes. The Bar charts show the concentration of SMRP expressed in nM, along with the standard error of the mean (SEM) (*p < 0.05, one-way ANOVA and Dunnett’s post-test). **(C,D)** bar charts showing the relative fluorescence units (RFU), along with SEM, of cells 72 h after being electroporated with vectors harboring the four haplotypic variants of MSLN promoter **(C)** or the rare variant for each of the considered SNPs (circled) **(D)** upstream of a GFP reporter. Since no statistically significant difference between the RFU of the two cell lines emerged from the mANOVA, the data from MeT-5A and Mero-14 have been combined (*p < 0.05, one-way ANOVA and Dunnett’s post-test).

Among the non-MPM volunteers, we found a statistically significant difference between the plasmatic levels of SMRP in heterozygotes H1H2, H1H3, and H1H4 compared with those of the reference group H1H1 (*p*-value ANOVA < 10^–5^). The Dunnett’s post-test showed a statistically significant difference between carriers of haplotype #2 or haplotype #3, but not haplotype #4, and the reference H1H1 (*p*-value < 10^–5^ and 0.0047, respectively) (Figure 2A). On average, carriers of haplotype #2 showed the highest SMRP level (average ± standard error: 1.30 ± 0.046 nM) followed by carriers of haplotype #3, #1, and #4 (0.98 ± 0.060 nM, 0.80 ± 0.022 nM, and 0.78 ± 0.047 nm, respectively). We did not observe any significant difference within the group of MPM patients (Figure 2B).

### *In vitro* Studies Confirm the Functional Role of Haplotype #2 and Identify rs2235503 C > A as the Most Likely Causative SNP

To evaluate whether the haplotype #2 and #3 could enhance the activity of the *MSLN* promoter, we carried out *in vitro* experiments using a fluorescence reporter assay. We transfected MeT-5A and Mero-14 cells with HR vectors carrying the GFP reporter gene under the control of the different haplotypic variants of *MSLN* promoter, named HR_HAP1, HR_HAP2, HR_HAP3, and HR_HAP4 (Figure 1A). Results showed a statistically significant difference among haplotypes but not between cell lines (mANOVA *p*-value = 0.0016 and 0.67). When compared to cells transfected with HR_HAP1, cells transfected with HR_HAP2 showed a 1.41-fold increased RFU (±0.267; *p*-value = 6 × 10^–4^). HR_HAP3 conferred a slight increased expression, but not statistically significant (1.07 ± 0.131; *p*-value = 0.45). HR_HAP4 showed a promoter activity similar to HR_HAP1 (0.97 ± 0.05; *p*-value = 0.98) (Figure 2C). Since haplotype #2 carries the uncommon variants of the four SNPs, we investigated the functional role of each of them, by employing four more constructs: HR_247, HR_246, HR_503, and HR_504 (Figure 1A). Each of these vectors bore the uncommon variant of rs3764247, rs3764246, rs2235503, or rs2235504, thus differing from HR_HAP1 only for one SNP. Again, mANOVA showed a statistically significant difference among genotypes but not between cell lines (*p*-value < 10^–5^ and 0.15). When compared with HR_HAP1, only cells transfected with HR_503 showed a significant RFU increase (fold change 1.35 ± 0.164; *p*-value < 10^–5^) (Figure 2D).

### Rs2235503 C > A Significantly Affects the Performance of SMRP as Diagnostic Biomarker for MPM

**FIGURE 3:**
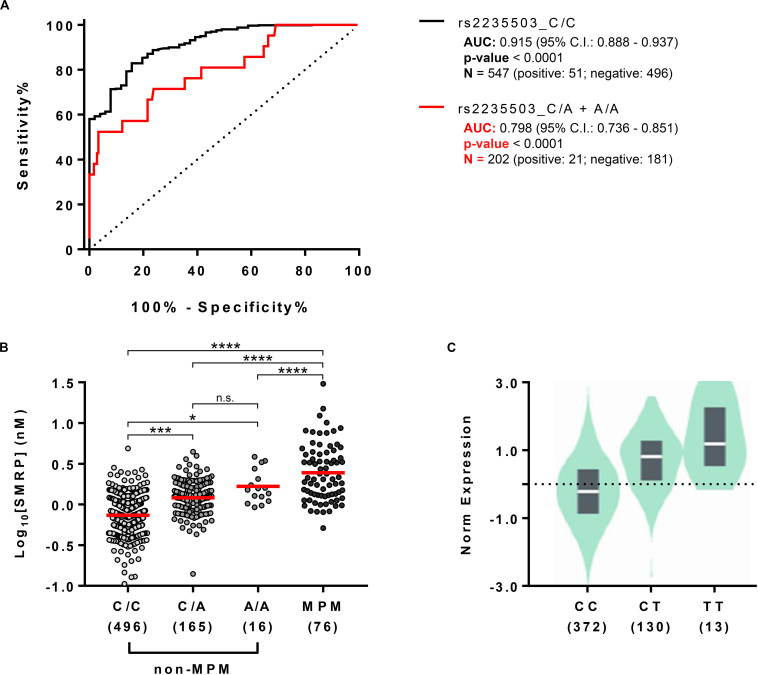
Effect of rs2235503 on SMRP and *MSLN*. **(A)** ROC curves showing the performance of SMRP as diagnostic biomarker for MPM in the subset of individuals carrying the common variant of the rs2235503 C > A (rs2235503_C/C) and in the subset of subjects carrying at least one rare variant of the same SNP (rs2235503 C/A + A/A). **(B)** Comparison between the plasmatic concentrations of SMRP in non-MPM subjects stratified based on their rs2235503 alleles, and in MPM patients. The dots represent the log_10_ [SMRP] (nM) of each subject. The red lines represent the median of the log_10_ [SMRP] (^*n.s.*^*p* > 0.05; *p < 0.05; ***p < 0.001, and ****p < 0.0001.) **(C)** Violin plot showing the normalized levels of MSLN mRNA in lung samples from 515 subjects stratified based on their rs12597489 C > T alleles, according to the GTEx Portal V8. Given the good linkage disequilibrium between the rs2235503 C > A and the rs12597489 C > T (*r*^2^ = 0.8), we considered this latter as a “tagging SNP” for rs2235503. According to GTEx, the minor allele of this SNP is associated with MSLN mRNA with a p-value of 1 × 10^– 49^ and a normalized effect size (NES) of 0.88.

Since the results indicated that the uncommon variant A-rs2235503 caused an increase in *MSLN* expression and SMRP levels in non-MPM subjects, we sought to determine whether this effect could significantly affect the accuracy of SMRP as a diagnostic biomarker for MPM. To verify this aspect, we stratified our cohort in two subgroups, the former containing the carriers of A-rs2235503 (rs2235503_C/A + A/A) and the latter containing all the other subjects (rs2235503_C/C). We then used the SMRP levels to calculate the “ROC” curve for each of the two subgroups. Results suggested a strong influence of the genotype on the performance of SMRP. In fact, the ROC curve for the C/A + A/A group resulted in an area under the curve (AUC) of only 0.798 ± 0.055 compared with an AUC of 0.915 ± 0.018 for the C/C group (Figure 3A). Interestingly, the AUC for the C/C group was also higher than that for the overall population (0.877 ± 0.019). Moreover, the Youden index pointed at an optimal cut-off value of 1.118 (sensitivity = 84.31, specificity = 82.86) for the C/C group, and 3.092 (sensitivity = 52.58, specificity = 96.69) for the C/A + A/A group. The optimal cut-off value for the overall population was 1.28, with a sensitivity of 77.78 and a specificity of 79.91. Notably, using a cut-off of 1.28 nM, the specificity for the C/C group raised to 88.71, but the sensitivity dropped to 76.47. Similarly, the specificity for the C/A + A/A group raised to 55.80, but the sensitivity dropped to 80.95. Supplementary Tables 2–4 report the complete list of the possible cut-off values along with the associated sensitivity and specificity for each group. These results were not surprising as the difference in the average SMRP concentration between non-MPM and MPM subjects, although still significant (*p*-value < 0.0001), dropped from 2.734 ± 0.174 nM for the C/C group to only 1.706 ± 0.388 nM for the A/A group (Figure 3B). Intriguingly, data from GTEx Portal (V8) showed a similar trend for the tagging SNP rs12597489 C > T, in linkage disequilibrium (LD) with rs2235503 (*r*^2^ = 0.8 according to HaploReg v4.1), suggesting a strong relationship between the mRNA levels of *MSLN* and the serum concentration of SMRP (Figure 3C). To explore the potential relationship between the rs2235503 and the prognosis of MPM patients, we carried out a survival analysis. The results showed a lack of association between this SNP and the overall survival (*p*-value = 0.66). The multivariate analysis performed to evaluate the prognostic significance of rs2235503, SMRP, and histology led to similar results, with none of these factors being significantly associated with prognosis (Supplementary Figures 1A,B).

## Discussion

Soluble mesothelin-related peptide is one of the most promising biomarkers for MPM, but its sensitivity and specificity are suboptimal for use in the clinical practice (Scherpereel et al., 2006; Casjens et al., 2017). Our previous data showed that SNPs within regulatory regions of the *MSLN* gene were associated with SMRP levels, raising the question of whether the evaluation of these variants could improve the performance of SMRP in the diagnosis of MPM (Garritano et al., 2014; De Santi et al., 2017). Herein, we explored the possibility of exploiting eQTL for *MSLN* to increase the diagnostic accuracy of SMRP. Firstly, we carried out an association study in a cohort of 677 non-MPM subjects and 72 MPM patients. We found that two haplotypes (#2 and #3) of the minimal promoter region of the *MSLN* gene were associated with increased serum levels of SMRP in non-MPM subjects. In agreement with our previous studies, we did not observe any association in the cohort of MPM patients (Garritano et al., 2014; De Santi et al., 2017). While we cannot rule out that the lack of association observed in MPM patients could be ascribed to a limited statistical power (*n* = 72), Goricar et al. (2020) recently reported similar results, showing that the minor allele of the rs1057147 was associated with increased SMRP levels in non-MPM (*n* = 782) but not in MPM subjects (*n* = 154). Together, these observations seem to corroborate the hypothesis that genotype has only a limited effect on SMRP levels in the malignant context, probably due to the major role played by other cancer-related factors in altering SMRP concentration. However, more studies on other cohorts of MPM patients are warranted for better exploring this effect among affected people (Pass et al., 2008). Nonetheless, the *in vitro* assays confirmed the functional role of haplotype #2, able to increase the expression of a reporter gene in a chimerized vector of about 42%. Moreover, cells transfected with a vector harboring the A-rs2235503 showed increased promoter activity of a similar extent, compared to C-rs2235503. This result straightened the role of haplotype #2 and pinpointed rs2235503 as the SNP responsible for the enhancement of *MSLN* gene expression. Since SMRP derives from the proteolytic cleavage of mesothelin (Sapede et al., 2008), it is conceivable that its plasmatic levels can be influenced by factors such as the eQTL for *MSLN*. Our work supports the rationale that the polymorphism rs2235503 is among these factors. Unfortunately, we could not perform any measurement of mRNA *MSLN* expression on the cohort of volunteers recruited in this study, however, indirectly, also the data from 525 lung samples within GTEx biobank confirmed a relationship between the genotype and the extent of *MSLN* gene transcription. Conversely, our *in vitro* studies ruled out the possibility of a functional role for the SNPs that characterize the haplotype #3 (i.e., rs3764246 and rs2235504). Thus, likely, the association observed *in vivo* should be ascribed to other SNPs in LD with haplotype #3 but residing outside the 1,000-bps promoter region herein considered. Since the results from other groups suggest the possibility of a prognostic significance of SMRP-affecting SNPs (Dipalma et al., 2011; Goricar et al., 2020), we tested whether genotype and SMRP levels could be predictive of a worse prognosis in our cohort. Our observations seem to rule out this hypothesis. Given the flaw association between SNP and SMRP among patients, and the lack of association between SMRP and overall survival, it is not surprising that there is a lack of significant relationship between SNP and patients’ survival in our cohort. However, given the limited number of MPM patients recruited in the present study, this result should be carefully evaluated and more work involving additional cohorts is needed to elucidate the prognostic role of the rs2235503. Then, we assessed whether the performance of SMRP could be improved when taking into account the subjects’ genetic background, in agreement with previous findings for other biomarkers (Cramer et al., 2003; Gudmundsson et al., 2010; Enroth et al., 2014; Ruggiero et al., 2015; Wang et al., 2018). Notably, we found that the AUC for individuals carrying the rs2235503-C/C genotype was higher (0.915) than that of carriers of the A-allele (0.798) and higher as compared to the whole population (0.877). Moreover, the Youden index pointed at significantly different cut-off values according to the rs2235503 genotypes, suggesting that the stratification of subjects based on this SNP could improve the accuracy of SMRP. In conclusion, our work shows that rs2235503 affects *MSLN* gene transcription and represents an eQTL for SMRP levels. Moreover, we highlighted for the first time that the rs2235503 can affect the diagnostic performance of SMRP, reinforcing the importance of considering the individual genetic background to improve the accuracy of cancer biomarkers (Cramer et al., 2003; Gudmundsson et al., 2010; Enroth et al., 2014; Wang et al., 2018). Therefore, it is conceivable that the characterization of further eQTL could help to translate SMRP into the clinical practice and improve the efficiency of this biomarker.

## Data Availability Statement

Publicly available datasets were analyzed in this study. This data can be found here: GTEx Portal (https://gtexportal.org/home/index.html) and dbSNP (https://www.ncbi.nlm.nih.gov/snp/).

## Ethics Statement

The studies involving human participants were reviewed and approved by Ethical Committee of the University Hospital of Pisa. The patients/participants provided their written informed consent to participate in this study.

## Author Contributions

SL and FG conceived the idea at the basis of the study, supervised the development of the work, and revised the manuscript. AB, RF, and AC from the Occupational Medicine of Cisanello Hospital in Pisa, contributed with essential biological samples and the related clinical features. CD carried out DNA extraction and genotyping. RS and PP obtained the plasmids via cloning and mutagenesis and performed the functional studies with the contribution of SM, AC, VN, ID, DM, EP, and MC. ME carried out the cytofluorimetric analyses. RS wrote the manuscript. PP, CD, VN, ID, DM, SL, and FG contributed to the revision of the manuscript. All authors contributed to the article and approved the submitted version.

## Conflict of Interest

The authors declare that the research was conducted in the absence of any commercial or financial relationships that could be construed as a potential conflict of interest.

## Footnotes

1 https://genome.ucsc.edu/

2 https://gtexportal.org/

3 https://www.internationalgenome.org/1000-genomes-browsers/
