# Supplementary material for: Variation rs2235503 C > A Within the Promoter of MSLN Affects Transcriptional Rate of Mesothelin and Plasmatic Levels of the Soluble Mesothelin-Related Peptide
Source: Front Genet. 2020 Aug 18;11:975. doi: 10.3389/fgene.2020.00975 (PMC7461867; doi:10.3389/fgene.2020.00975)
Supplement: Supplementary file 1 [file Data_Sheet_1.DOCX]

| **SNP** | **p-value** | **Effect size** | **Other SNPs in linkage disequilibrium** |
| --- | --- | --- | --- |
| rs2235503 | 4.4x10^-33^ | 0.98 | rs12597489 (r^2^=0.80) |
| rs2235504 | 3.6x10^-27^ | 0.81 | rs3764246 (r^2^=0.94)  rs2235505 (r^2^=0.92) |
| rs3764246 | 7.5x10^-27^ | 0.80 | rs2235504 (r^2^=0.94)  rs2235505 (r^2^=0.90) |
| rs3764247 | 7.5x10^-25^ | 0.79 | - |

**Supplementary Table 1:** Association between the four selected SNPs and the MSLN mRNA levels in lung tissues according to GTEx v6.
