# Supplementary material for: Variation rs2235503 C > A Within the Promoter of MSLN Affects Transcriptional Rate of Mesothelin and Plasmatic Levels of the Soluble Mesothelin-Related Peptide
Source: Front Genet. 2020 Aug 18;11:975. doi: 10.3389/fgene.2020.00975 (PMC7461867; doi:10.3389/fgene.2020.00975)
Supplement: Supplementary file 5 [file Data_Sheet_5.DOCX]

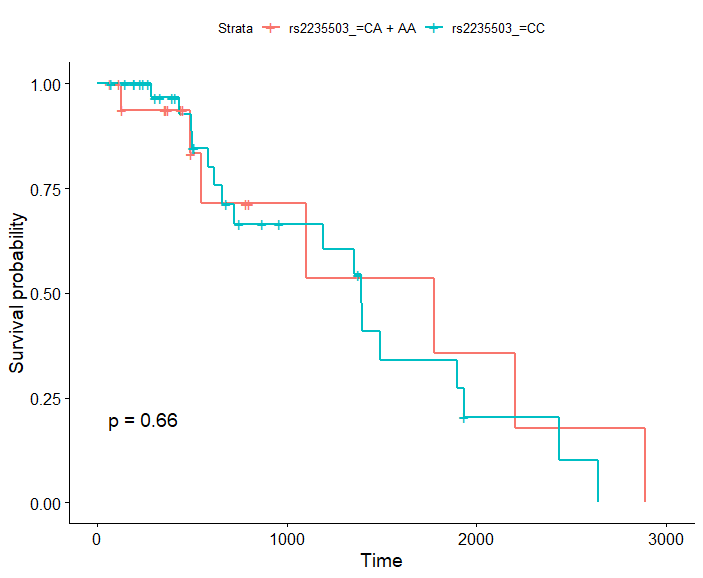


**Supplementary Figure 1A:** Kaplan-Meier showing the relationship between rs2235503 and the overall survival in 57 MPM patients of our cohort. The plot represents the survival probability of the homozygotes for the common allele (CC) against the carriers of at least one rare variant (CA + AA), along with the p-value.


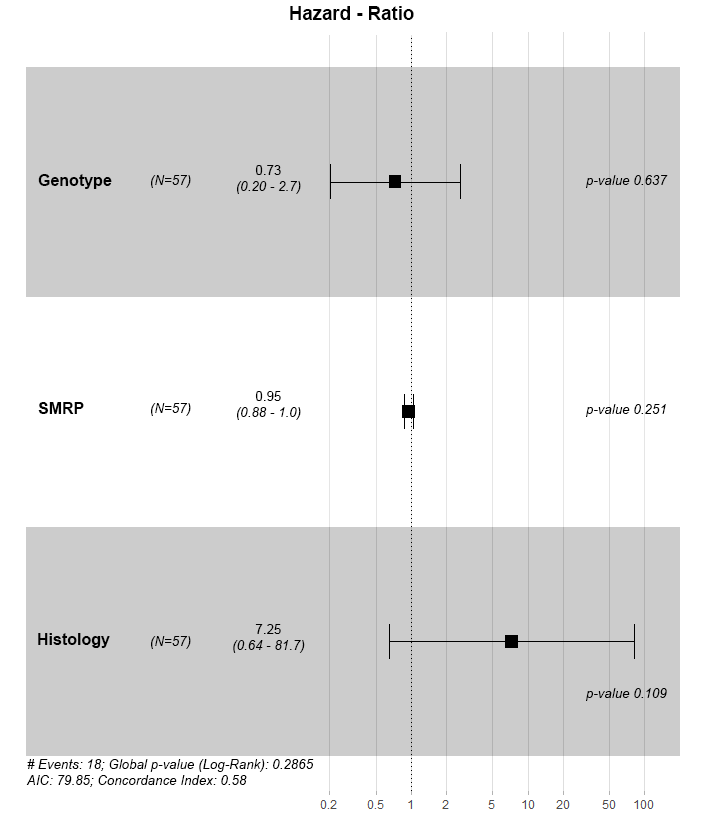


**Supplementary Figure 1B:** Forest plot showing the results of the multivariate analysis performed to assess the prognostic significance of SMRP, Genotype, and Histology. The graph shows average hazard ration along with the 95% confidence intervals and the p-value for each variable.
